# Protein designer David Baker: I like doing things that seem like magic

**DOI:** 10.1093/nsr/nwaa071

**Published:** 2020-04-17

**Authors:** Weijie Zhao, Chu Wang

**Affiliations:** NSR news editor based, Beijing; Professor at Peking University, Beijing

## Abstract

Search ‘*de novo* protein design’ on Google and you will find the name David Baker in all results of the first page. Professor David Baker at the University of Washington and other scientists are opening up a new world of fantastic proteins. Protein is the direct executor of most biological functions and its structure and function are fully determined by its primary sequence. Baker's group developed the Rosetta software suite that enabled the computational prediction and design of protein structures. Being able to design proteins from scratch means being able to design executors for diverse purposes and benefit society in multiple ways. Recently, *NSR* interviewed Prof. Baker on this fast-developing field and his personal experiences.

## WHY AND HOW TO DESIGN PROTEINS?

**NSR:** You have talked about the ‘protein-design revolution’ many times. What are the missions of this revolution?

**Baker:** It is a technological revolution like other technological revolutions in human history. We are learning how to control the world in new ways. When people learnt to control metal, we went out of the Stone Age. And, in the Industrial Revolution, people learnt how to control things using steam engines. After that, there was the digital revolution that enabled us to store information digitally in computers. And now, with this ‘protein-design revolution’, we are learning how to control biomolecules in ways that were not possible before.

**NSR:** So we are going to make tools with proteins?

**Baker:** Yes. We are going to be able to make things out of proteins in ways that we never could before. For example, we can make new types of drugs, vaccines, therapeutics and even materials.

**NSR:** Both protein-design and protein-structure prediction are based upon the protein-folding principles. Have we fully understood these principles?

**Baker:** We understand these principles in the broad outlines, but we do not understand all the details yet. We understand that proteins generally fold to their lowest energy states. We understand how to calculate energies reasonably well. But we still cannot do that as accurately as we need for very precise functions.

I believe there will be further theory progressions. And we will also get more feedback from the experiments. Those efforts will combine to improve our description of atomic information.

**NSR:** Now, in your lab, what is the common procedure to design a new protein with a certain function?

**Baker:** It depends on what that function is. For example, if you want to make a protein that binds to a cancer-cell target, you would first identify the binding surface, which requires a little bit of understanding about the structure and the biology of the target. Then you would make a candidate protein with a rough shape complementarity to the target and further design the sequence on its binding interface so that it can geometrically and chemically engage with the target. After that, you would check

**Figure 1:**
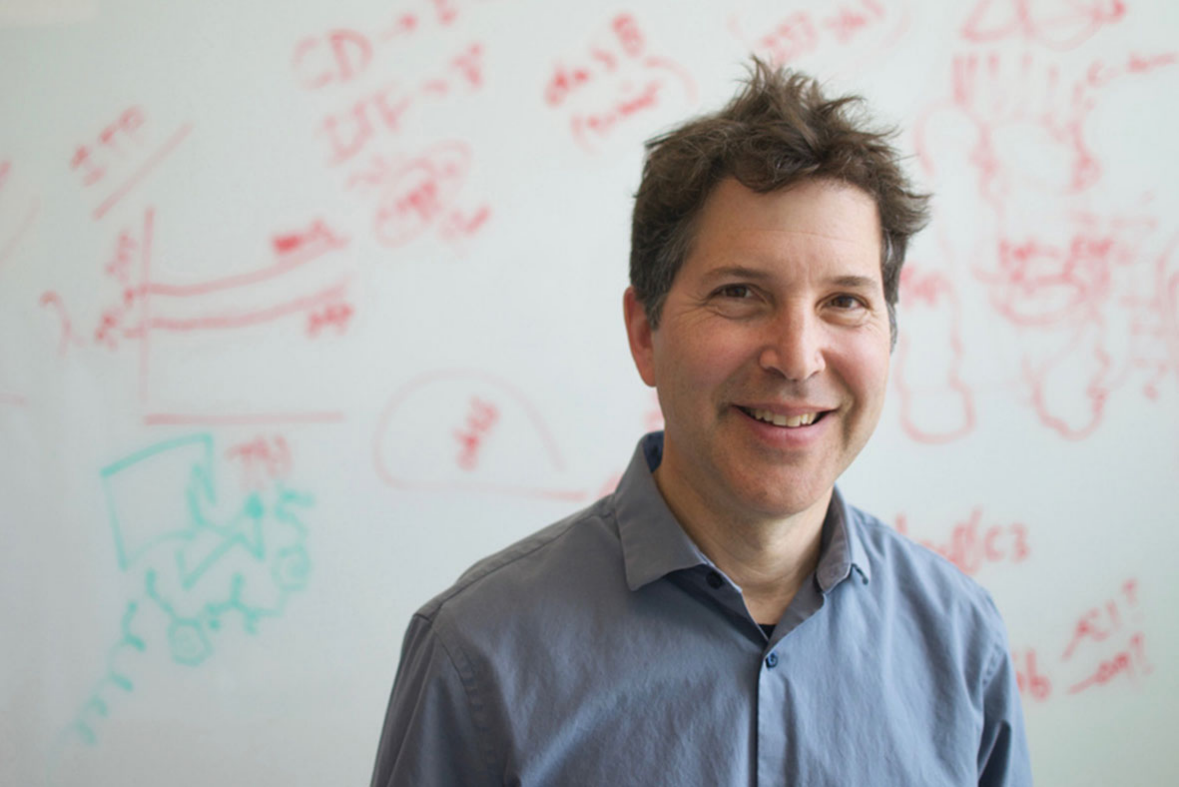
Professor David Baker at the University of Washington is a world leader of protein design (*Courtesy of Prof. Baker*).

the new sequence that you had designed to see whether that designed structure is in the lowest energy state of that sequence. These steps can be basically done on the computers automatically.

And then you would make a synthetic gene encoding that brand new designed structure and put it into bacteria cells to make the protein so that you can purify the protein and see whether it binds to the target. Recently, we have worked out methods for testing tens of thousands of designs all in parallel to figure out which ones can bind the target.

**NSR:** If we ask two researchers to design the same thing, will they achieve the same structure?

**Baker:** The space of possibility is very big. There are stochastic parts in the algorithm and the computer makes random choices. So, even if you run the same computer program twice, you probably would not get exactly the same results.

**NSR:** How long would it take to design a protein?

**Baker:** It would not take too long to make a design on the computer. Typically, you would not just make one; you would make hundreds of thousands of models. And then you would filter them down based on various criteria to pick those to be actually tested.

What actually takes the time is after that. It will take 4–6 weeks to do all the experiments. After getting the results, you will often need to go back and update the designs based on those results. It is not one single design cycle. This iterative process is what takes a long time. Through this repeating process, we also learn how to do it better. So what we are doing is more science than engineering.

**NSR:** What is the success rate?

**Baker:** The success rate is a function of time depending on the problem that we are trying to solve. Typically, when we pick a new problem, the success rate will be low. And, when we have found a way to conquer that problem, the success rate will be generally higher.

## A ZOO OF MAGICAL PROTEINS

**NSR:** Many of your designed proteins are formed of alpha helixes. Why?

**Baker:** I think we started with alpha helixes because the interactions stabilizing the helixes are between amino acids closer in the primary sequence. It is more complicated to figure out how beta strands come together to form beta sheets.

But now our understanding of the principles for beta-sheet proteins has improved a lot, and it is no longer a problem. We are now routinely making proteins out of only beta sheets. For example, we designed beta-barrel proteins that go through the membrane and let ions through.

**NSR:** One of your designed barrels shows selectivity for potassium-ion transfer. Is it designed to be so?

**Baker:** No. We were just trying to design proteins with pores of different sizes and we were surprised that it was selective for potassium. People have been arguing about how potassium channels work and our work showed that maybe it is just the right size for potassium ions with the water molecules being stripped off. So that is an example of how protein design can help to better understand how natural proteins work.

**NSR:** You designed a protein rotator assembled with two independently designed parts. So is protein design becoming modular?

**Baker:** That's right. We are making a common set of building blocks that can be easily put together in different ways for *de novo* protein design.

**NSR:** DNA/RNA design is also developing fast. Will protein design combine with the design of DNA/RNA or other materials?

**Baker:** Yes, it will be very interesting to combine protein design with DNA origami. We are also designing proteins interacting with inorganic compounds. For example, we are trying to control inorganic crystal growth with proteins. This may be helpful for applications including semiconductor production, etc.

**NSR:** Are your designs beginning to be applied in translational science?

**Baker:** Yes. We are starting several companies to commercialize these protein designs. There is a company called Neoleukin Therapeutics, which is trying to commercialize the designed protein mimicking interleukin-2 for cancer treatment. We also have a company that is developing vaccines for various types of viruses.

**NSR:** What are the proteins you are working on now?

**Baker:** We have quite a few interesting directions. We are working on transmembrane pores, which may have many applications such as filtering and nanopore DNA sequencing. We are also trying to design molecular machines, new types of drug molecules and different types of materials. In addition, we want to design proteins that are able to regulate cell behaviors such as cell growth, cell differentiation and cell fate.

**NSR:** To many people, work from your lab seems like magic.

**Baker:** Sometimes it seems like magic to me, too. I like doing things that seem like magic. But I should point out some kind of dishonest or misleading impression here. We show them the successful magic-like designs but seldom talk about all the steps and the things that did not work.

We show them the successful magic-like designs but seldom talk about all the steps and the things that did not work.

—David Baker

## FOLD IT UP ON YOUR LAPTOP

**NSR:** How did you come up with the idea of Foldit, the online puzzle game about protein folding and design?

**Baker:** We were doing protein-structure prediction and it required a lot of computers. While we kept getting more computers, it was expensive and we soon ran out of room. So we started a project called Rosetta@home to get volunteers around the world to help with our calculations on their own computers. It was a screen saver showing how the protein is folding up on the screen as the computer is doing the calculation.

Then we began to get letters from these volunteers saying that they did not think the computer was doing a very good job of folding up the proteins and they themselves could do a better job. So, one day, when I was hiking with the father of a friend of my daughter, who is a computer scientist, we talked about this and came up with the idea of making a game out of Rosetta@home, which became Foldit later.

**NSR:** How is Foldit going? Will it change in the future?

**Baker:** It is going very well. We have a lot of people who have signed up and several hundred people online are playing at the same time. There has been a *Nature* paper demonstrating the proteins designed by Foldit players.

Foldit started out first to do protein-structure prediction and now it is for protein design. We will continue to release new puzzles for the players to solve. So it will keep changing as our research interest changes.

## BUILD UP THE PROTEIN-DESIGN COMMUNITY

**NSR:** Are there other good protein-design groups besides your group?

**Baker:** There are many protein-design labs. Different labs have different interests and are doing different kinds of work. I am really interested in *de novo* protein design personally. But, if you want to solve a specific application problem, modifying a naturally occurring protein may be a better choice than building a new protein from scratch.

**NSR:** You have trained a large number of scientists around the world.

**Baker:** Many protein-design scientists around the world have once come through my group. We started a Rosetta community called Rosetta Commons, which is now a big collaboration including many universities and institutions. Many groups are communicating and collaborating to improve the Rosetta program within this community and this has been very fruitful.

Now there are many companies using the Rosetta program. They need to license it and pay a fee every year. But I decided at the beginning that no one would make any money personally from the Rosetta program. So that money, more than 1 million dollars a year, was used to support various activities of Rosetta Commons.

We have a RosettaCon conference every year. Everyone comes back to talk about their latest results and we together decide what to do with the program. It is a big international and collaborative effort now and we are going to have two meetings a year starting from 2019. We also use that money to run training camps for people who are interested in Rosetta but do not know how to use it. Work of the new students will help the Rosetta community. It is great to be able to collaborate with everyone.

## BAKER’S STORY

**NSR:** How did you begin to use computers for biological research?

**Baker:** My graduate work was entirely experimental. I felt like learning something new and the first day on which I started my Postdoc, there was a computer on my desk. I needed to do some work on crystal graphic refinement with it.

My Postdoc lab was a structural-biology lab. There was a room where everyone was solving crystal structures by sitting at the computer terminal and tracing the amino-acid chain to the electron density. I tried doing that for 3 minutes and got a bad headache. This makes me realize that this type of computational work was impossible for me to do and I would like to try something different.

**NSR:** When did you begin with protein-structure prediction? Did you write it in your job proposal for the University of Washington?

**Baker:** No, my job proposal was pretty much experimental as well. But I had been interested in protein folding for a long time and I suggested that my second student should think about protein-structure prediction. I usually tell people to focus, but this time I did tell him to do things so completely differently. I don’t know why.

**NSR:** What was your expectation when you started to research on protein folding? Did you imagine that now you can design proteins from scratch?

**Baker:** I did not have any expectation. Actually, I have an idea of what is going to happen in 6 months from now, but I do not plan too far ahead. If you can plan it, it is not science. You always hope to discover something you were not able to imagine.

**NSR:** It is said that you are still doing your own project.

**Baker:** I was busy in the last couple of months. But yes, I still do my own project and present my own research at the group meeting. I do not feel nervous when I give a talk at a big conference because I am not going to see the audience again for a while. But I get really nervous when I have to present at the group meeting, especially in front of the people I work with who are real experts on the subject.

If you can plan it, it is not science.

—David Baker

**NSR:** That is really valuable. Most of the well-known scientists do not do that.

**Baker:** For me, it really started after my kids left home so that I have a lot of time. If I am starting a project, it should be something completely new. Once, at a RosettaCon, I talked much about the helix-bundle proteins. Everyone got tired of me, but 2 years later, there was a whole session of people working on it.

**NSR:** Please give some advice to young researchers. How can you be so creative and publish so many top articles?

**Baker:** I think the most important thing is to pick big and challenging problems. You can choose to work on problems that are

completely new and unsolved or problems that are sort of being solved. But, whatever you choose, it will always be hard; there will be a lot of work to do anyway. So do not be too worried about whether things are safe. Just pick the big problems and enjoy your work. Once you solve them, you will advance science and there will be major publications.

